# Ultra-Wideband and Wide-Angle Microwave Metamaterial Absorber

**DOI:** 10.3390/ma11102045

**Published:** 2018-10-20

**Authors:** Xavier Begaud, Anne Claire Lepage, Stefan Varault, Michel Soiron, André Barka

**Affiliations:** 1 LTCI, Télécom ParisTech, Université Paris-Saclay, 46 rue Barrault, 75634 Paris CEDEX 13, France; Anne-Claire.Lepage@telecom-paristech.fr (A.C.L.); stefan.varault@yahoo.fr (S.V.); 2 SART, 16 Allée des Quatre-Coins, 91190 Gif-sur-Yvette, France; Michel.Soiron@sart.fr; 3 ONERA/DEMR, Université de Toulouse, 31000 Toulouse, France; Andre.Barka@onera.fr

**Keywords:** electromagnetic wave absorbers based on multilayer structures, metasurface, ultra-wideband microwave absorber, metamaterial absorber, frequency selective surface, wide-angle metamaterial absorber, wide-angle impedance matching layers, self-complementary structures, anti-phase metasurface

## Abstract

In order to extend the performance of radar absorbing materials, it is necessary to design new structures with wideband properties and large angles of incidence which are also as thin as possible. The objective of this work, realized within the framework of the SAFAS project (self-complementary surface with low signature) is, then, the development of an ultra-wideband microwave absorber of low thickness. The design of such material requires a multilayered structure composed with dielectric layers, metasurfaces, and wide-angle impedance matching layers. This solution has been realized with on-the-shelf materials, and measured to validate the concept. At normal incidence, the bandwidth ratio, defined for a magnitude of the reflection coefficient below −10 dB, is 4.7:1 for an absorber with a total thickness of 11.5 mm, which corresponds to λ/7 at the lowest operating frequency. For an incidence of 60°, this bandwidth ratio is reduced to 3.8:1, but the device remains ultra-wideband.

## 1. Introduction

Whether for civil or military applications, electromagnetic microwave absorbers or radar absorbing materials (RAM) have been undergoing many developments in recent years, to minimize interference issues between antennas on platforms or to improve the stealth of defense systems. Indeed, absorbing materials are usually used to reduce the reflection of electromagnetic waves on a surface. For space applications, these materials may, for example, be placed on the satellite to reduce interference between antennas. For airborne applications, the absorbers can also be mounted on the surface of objects to reduce their radar cross-sections. In antenna design, they can be used to suppress the radiation in a certain direction with an absorbing cavity. The solution presented in this article may be used for all these applications.

A conventional method for designing an absorber is to insert losses on the surface of the material. The Salisbury screen [1] is an example of this approach, in which a resistive layer with impedance equal to Z_0_ is placed over a metal surface at a distance equal to a quarter of the wavelength. The major disadvantage of this resonant structure is its narrow bandwidth operation. The Jaumann absorber [2], consisting of several resistive layers spaced approximately by a quarter wavelength, operates over a wider bandwidth. However, this technique greatly increases the thickness of the structure. In 2002, Engheta proposed to introduce metamaterials in the design of absorbers [3]. This approach has represented a technological breakthrough, as it allows reducing drastically the thickness, but only for a narrow bandwidth. This kind of absorber can be realized with the use of a high impedance surface (HIS) associated with a resistive material.

Metamaterial absorber is an inherently resonant structure, which implies a narrow operating bandwidth. The absorption level can be increased by superimposing the resonators as in the work of Landy and al. [4], but at the expense of the thickness and on a reduced bandwidth. One of the major challenges is, therefore, to increase this bandwidth. A simple and effective way to increase the absorption band from resonant structures is to create multiple resonances using different resonators that can be distributed in the same plane [5,6] or stacked vertically [7]. Wideband behavior is difficult to obtain for [5,6], but this solution is very thin. With the solutions [7], a wideband absorber is obtained, but it is at the expense of the thickness. The solution proposed in [3] is then a good alternative to increase the absorption. The metamaterial associated with a resistive material consists of a periodic array of printed patterns loaded with resistors, resistive sheets, or resistive inks, in order to achieve absorption [8], and placed over a grounded dielectric slab. By stacking a succession of dielectrics and resistive Frequency Selective Surfaces (FSS) it is possible to realize ultra-wideband absorbers [9,10,11,12]. This method makes it possible to obtain an ultra-wideband absorber, but at the expense of a greater thickness. However, their ease of manufacture and their low cost are advantageous. This compromise bandwidth versus thickness is well described in the work of Costa and al. [8]. Increasing the thickness is an issue for many applications where the space available to place the RAM is reduced. This increase of thickness is also often followed by an increase in weight, and becomes troublesome especially for airborne structures. In addition, increasing the thickness of the absorber also makes it more sensitive to incidence and polarization. Some solutions exist to make the absorber less sensitive to the incidence and polarization of the wave. The first is to introduce, into the structure, vertical metal strips or vias [13,14]. This solution is powerful, but introduces complexity into the structure. Another solution is to work on the symmetries of the unit cell [15,16]. It is also possible to use, like in antenna array design, a wide-angle impedance matching layer (WAIM) to minimize the impedance discontinuity between the absorber and free space and, thus, to improve the performance in terms of the angle of incidence [17,18]. However, again, this WAIM layer increases the thickness of the absorber material.

The geometry of the cell and its symmetries are of great interest, to minimize the dependence of absorption as a function of incidence, as we have just seen before. It can also increase the bandwidth by minimizing the reflected wave in the normal direction [19,20]. These anti-phase metasurfaces are composed of two different elements with 180° reflection phase difference, as in a chessboard-like configuration, to minimize the reflection. Moreover, when the unit cell is self-complementary like a chessboard, it can be used to enlarge the bandwidth with the help of the Babinet principle, like in wideband antenna design [21].

In this paper, we present the development of an ultra-wideband and wide-angle metamaterial absorber, developed within the framework of the SAFAS project (self-complementary surface with low signature). The innovative nature of this project is based on the combined use of a self-complementary structure, a metasurface, and a WAIM layer, to make possible a dual polarized self-complementary connected array antenna [21] and, also, an ultra-wideband and wide-angle metamaterial absorber. This combination of self-complementary structure, metasurface, and WAIM layer, has not been used in the previous cited works [3,4,5,6,7,8,9,10,11,12,13,14,15,16,20], and our objective is to optimize them together in order to obtain an ultra-wideband and wide-angle metamaterial absorber of low thickness.

## 2. Materials and Methods

This design is directly inspired by the methodology used in the first phase of the project, which focused on an ultra-wideband dual-polarized antenna with low RCS [21]. The work is carried out in several steps, the first of which uses a pre-dimensioning and optimization tool using transmission line (TL) model [22], followed by full-wave simulation within the assumption of infinite arrays. The two last steps are the realization and the validation with the measurement.

**Figure 1:**
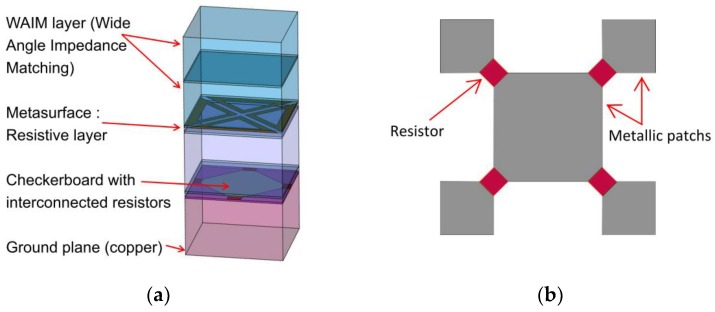
(**a**) Multilayer absorber description (unit cell); (**b**) Unit cell of the checkerboard with interconnected resistors.

The architecture of the absorber material that has been optimized [23] is presented in Figure 1a. The lower part is the self-complementary structure (checkerboard) which inherently has a wideband behavior. The elementary cell of the checkerboard is given in Figure 1b, where the metallic parts are drawn in grey. To achieve the absorption of the structure, resistive deposits were placed between each conducting patch of the checkerboard. This self-complementary structure is placed above a ground plane. To increase the bandwidth of the structure, a metasurface, also using resistive deposit, has been optimized and is located above the checkerboard. Finally, to minimize the impedance discontinuity between the absorber and free space, and to provide wide-angle and wideband frequency matching, a WAIM layer has been added at the top of the structure.

**Figure 2:**
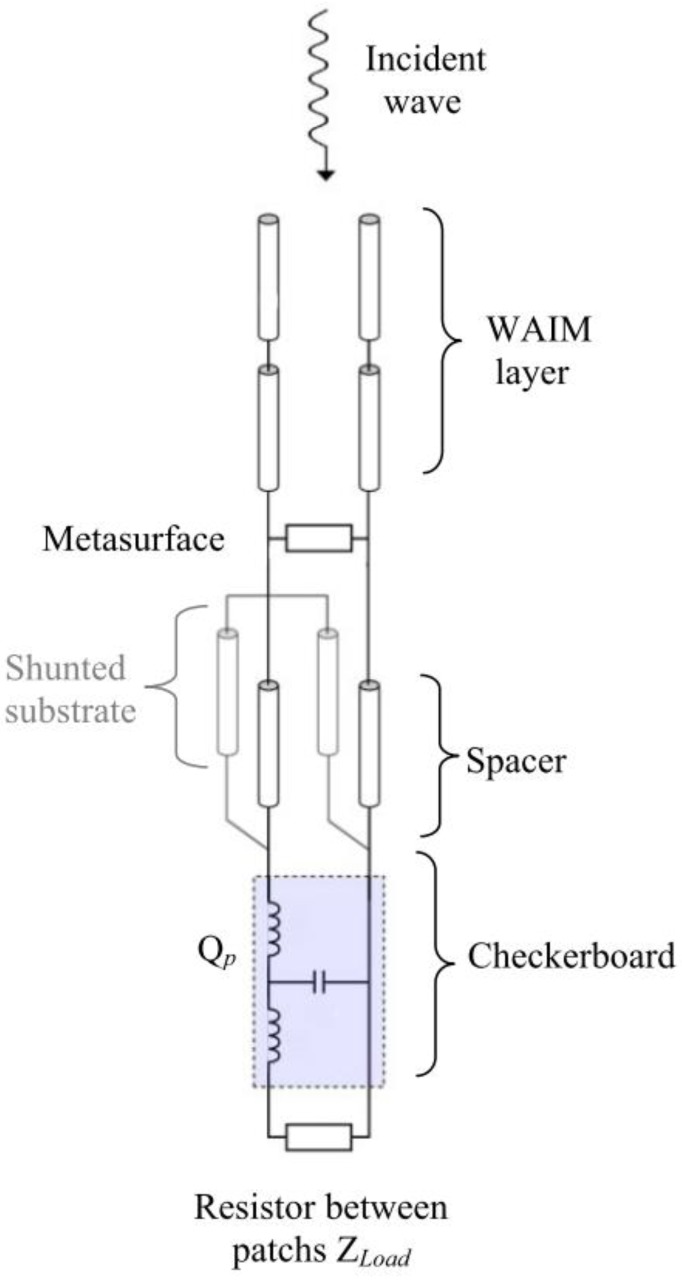
Transmission line (TL) model of the absorber corresponding to Figure 1a.

We have used these principles to build a fast and accurate TL model of the absorber, which is presented in Figure 2.

The whole absorber can conveniently be modeled by using the transmission line formalism [22], where each dielectric layer can be described by a known ABCD matrix. The complete model of the multilayer is then obtained by cascading these different matrices [22]. The equivalent TL model of the absorber of Figure 1a is given in Figure 2.

This TL model, initially defined to evaluate the antenna’s performances in receive mode, was presented in detail in [21,22]. In this configuration, the feeding system of the antennas was replaced by equivalent impedance Z_Load_, and the performance of the antenna was highlighted by the calculation of its radar cross-section (RCS). This multilayered antenna was also composed with a bed of nails, in order to improve the RCS at lowest frequency in TM polarization and a double layer WAIM. In the present paper, related to the design of the absorber, as the constraints are different, some simplifications have been proposed. Indeed, the metasurface has become resistive, enabling suppression of the bed of nails, and keeping only one WAIM layer. In the TL model of the absorber, the impedance, Z_Load_, is a parameter used to improve the absorption level of the global structure.

All the dielectric layers are represented by transmission lines, and the metasurface is assumed to be homogenous and modeled by a surface impedance. The ABCD matrices of the transmission lines and the parallel impedance being well known, the major difficulty, therefore, comes from the modelization of the checkerboard. The accuracy of the results rely directly on that of the model of the resistive metasurface, or checkerboard loaded with resistors (Figure 1b). A previous study [21] has made it possible to extract a circuit model for the checkerboard of Figure 1b, loaded by any Z_Load_ impedance. This model is represented by the quadrupole, Qp, in Figure 2. This TL model leads to negligible calculation times and, thus, allows optimization of a large number of simultaneous parameters. By simulating this circuit with ADS [24], we can also benefit from the performance of the associated optimizer.

We have been able to optimize the absorber for both TE and TM polarizations, and for angles up to 60°, and on ultra-wide frequency bands, which would have been much more complicated with conventional global modeling software. After optimization, the size of the cell is 5 mm × 5 mm, and the total thickness is 11.5 mm. The following step is a full-wave simulation of the infinite array using the frequency domain finite element solver of CST Microwave Studio (CST MWS) with Floquet boundary conditions [25].

**Figure 3:**
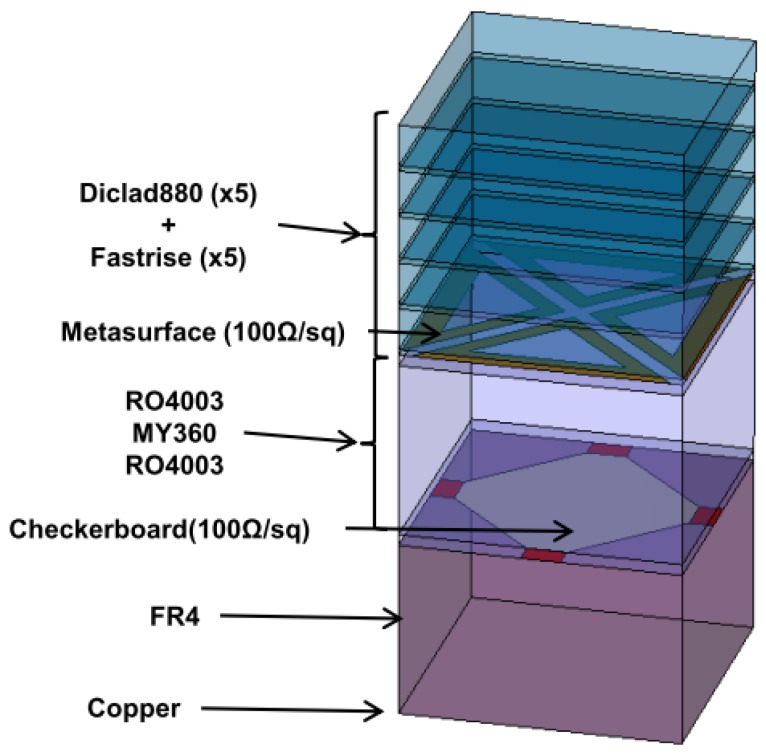
Description and nomenclature of the different layers of the ultra-wideband absorber.

In order to realize a prototype, after inventorying the available materials at the lab, we opted for the bill of materials given in Figure 3.

This choice was justified by the materials readily available at the time of the study. The absorber is composed with copper as ground plane, epoxy FR4 (glass-reinforced epoxy laminate, ε_r_ = 4.7, tan δ = 0.02), RO4003 (woven glass reinforced hydrocarbon/ceramics, ε_r_ = 3.38 and tan δ = 0.0027) from Rogers, MY360 (woven glass PTFE, ε_r_ = 2.33 and tan δ = 0.0011) from Neltec and Diclad 880 (fiberglass-reinforced PTFE-based composites, ε_r_ = 2.2 and tan δ = 0.0009) from Rogers. The checkerboard is constituted by metallic patches etched on RO4003 material, and connected at their corners with resistor foil NiCr (100 ohms per square) from Ticer Technologies. The metasurface is composed with the same resistor foil NiCr (100 ohms per square) etched on the second RO4003 material. All layers are finally connected together with a FASTRISE glue layer (ε_r_ = 2.7).

Other material choices were possible, but not reachable in the timeline of the SAFAS project. It is, therefore, not an optimum but a compromise to validate a proof of concept.

## 3. Results

**Figure 4:**
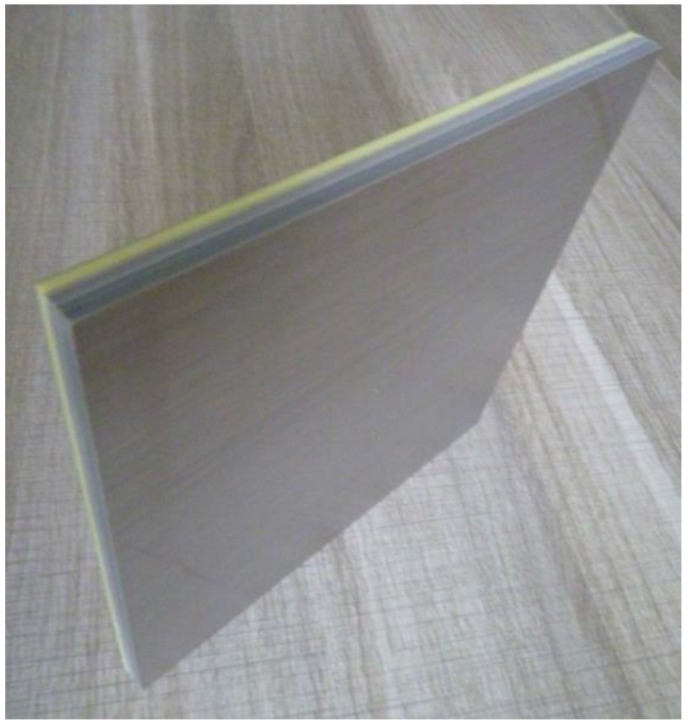
Prototype of the manufactured absorber.

The prototypes having the following dimensions (200 mm × 200 mm) and composed of 1600 cells were manufactured by CIRETEC (Figure 4).

The measurements were carried out in our laboratory with a bi-static characterization set-up. To perform measurements, we use the arch method measurement procedure to obtain RF absorber reflectivity [26,27]. This method consists in using two antennas. The transmitting antenna illuminates the absorber material, and the reflected wave is collected by the receiving antenna. The antennas are placed along the arch according to incidence angle (θi = θr), and the absorber material is placed at the center of arch. The detailed measurement process is given in [27].

**Figure 5:**
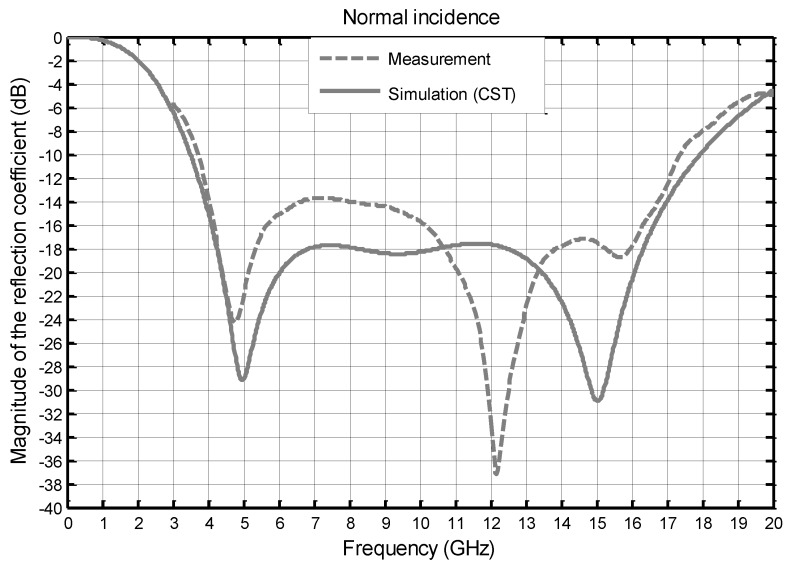
Comparison between the simulated and measured magnitudes of the reflection coefficient (dB) as a function of frequency at normal incidence.

**Figure 6:**
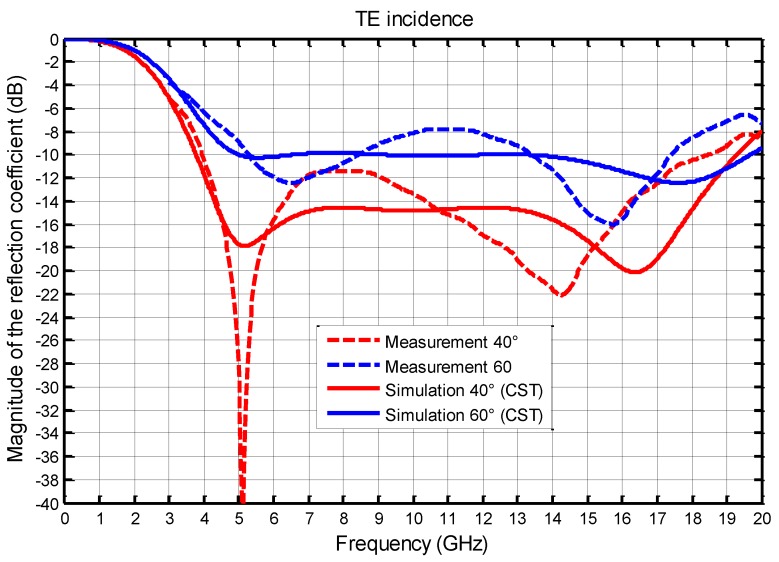
Comparison between the simulated and measured magnitudes of the reflection coefficient (dB) as a function of frequency with two angles of incidence 40° and 60° for TE polarization.

**Figure 7:**
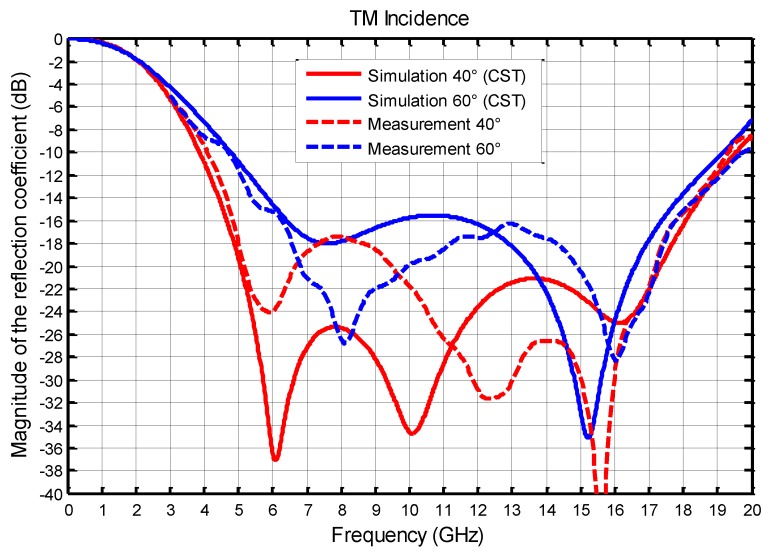
Comparison between the simulated and measured magnitudes of the reflection coefficient (dB) as a function of frequency with two angles of incidence 40° and 60° for TM polarization.

We present, in the following figures, the comparison between the measurements and the simulations (infinite array) at normal incidence (Figure 5) then, in TE (Figure 6) and TM (Figure 7), polarization for two angles of incidence 40° and 60°.

## 4. Discussion

At normal incidence (Figure 5), the simulation results give a bandwidth (−10 dB) of 3.5–18 GHz (5.1:1), while the measurement results give 3.7–17.5 GHz (4.7:1). These results seem different from each other, but the behavior of the absorber is well observed on a very large bandwidth. The level of the two characteristic minima, appearing at 5 GHz and 15 GHz in the simulation, is shifted around 4.7 GHz and 12 GHz in the measurements. This frequency shift, which will also be found in the other results, can be explained by two potential causes. The first one is a poor estimation of the dielectric constants. The FR4 material is a low-cost material, and the dielectric constant is subject to significant fluctuations. The second is probably due to the finite dimensions of the absorber. The level of the magnitude of the reflection coefficient between these two minima is higher in measurements than in simulations, and a poor estimation of the dielectric constant can also be the cause. Finally, it should be noted that these differences occur between −14 dB and −18 dB, which are intrinsically low values.

Comparisons between measurements and simulations results for 40° and 60° incidence are plotted in Figure 6 for TE polarization, and in Figure 7 for TM polarization. The bandwidth obtained at normal incidence is slightly reduced with TE and TM polarization, but remains very wideband. For TE polarization, at 5 GHz, the magnitude of the reflection coefficient is very different between simulation and measurement, but it should be noted that this great difference is between −18 dB and −40 dB, which are very low values and, then, not significant.

In simulation, at 40°, the bandwidth is 3.8–19.3 (5.1:1) and, at 60°, it is reduced to 5–19.6 GHz (3.9:1). For the measurement, the bandwidth is 4–18.5 GHz (4.6:1) at 40°, and is reduced to 5–19 GHz (3.8:1) at 60°. We find, quite well, the expected behavior of the absorber, despite differences between measurements and simulations of the same order as those found for the normal incidence. These results also clearly show a significant decrease in expected performance for an incidence greater than 40 degrees. This phenomenon, due to the diffractions by the edges of the absorber, is particularly marked when the incident electric field is parallel to the edges of the absorber (Figure 6).

## 5. Conclusions

The ultra-wideband and wide-angle metamaterial absorber, with a total thickness of 11.5 mm, developed during the SAFAS project, has been realized and measured to validate the concept. It was manufactured with materials available on the shelf and it is, therefore, usable for any applications compatible with their physical characteristics. Moreover, it can be noted that this type of structure can easily be modified to adapt the absorption performance to particular needs, and we have further shown that it was possible to produce ultra-wideband absorbers without using lossy materials. The thickness of the total structure could be reduced, but at the cost of a bandwidth reduction.

The measured performance of the prototype is as follows: at normal incidence, a bandwidth (−10 dB) of 3.7–17.5 GHz (4.7:1) is observed. This bandwidth ratio is slightly reduced at oblique incidence in the TE and TM polarizations but remains very broadband. At 40°, the bandwidth is 4–18.5 GHz (4.6:1) and at 60° it is reduced to 5–19 GHz (3.8:1).
